# Evaluation of treatment with two weeks of doxycycline on macrolide-resistant strains of *Mycoplasma genitalium:* a retrospective observational study

**DOI:** 10.1186/s12879-021-06910-1

**Published:** 2021-12-07

**Authors:** M. Gossé, S. A. Nordbø, B. Pukstad

**Affiliations:** 1 Department of Clinical and Molecular Medicine, Faculty of Medicine and Health Sciences, Norwegian University of Science and Technology, Postbox 8905, 7491 Trondheim, Norway; 2 Department of Medical Microbiology, St. Olav’s Hospital HF, Trondheim University Hospital, Trondheim, Norway; 3 Department of Dermatology, St. Olav’s Hospital HF, Trondheim University Hospital, Trondheim, Norway

**Keywords:** *Mycoplasma genitalium*, Doxycycline, Drug resistance, Macrolide resistance

## Abstract

**Background:**

Increasing macrolide resistance makes treatment of *Mycoplasma genitalium* infections challenging. The second-line treatment is moxifloxacin, an antibiotic drug best avoided due to the potential of severe side effects and interactions. This study evaluates the effects of treatment with doxycycline 100 mg twice daily for 2 weeks as an alternative to moxifloxacin.

**Methods:**

This retrospective observational study examined the medical records of patients testing positive for macrolide resistant *Mycoplasma genitalium* from January 1st, 2016 to September 1st, 2019 in Trondheim, Norway. Information regarding symptoms as well as clinical and microbiological cure was collected.

**Results:**

263 infections from 259 patients (161 females/98 males) were examined. 155 (58.9%) had a negative test of cure following treatment. 34.7% of symptomatic patients not achieving microbiological cure experienced symptom relief or clearance. There was no statistical difference between bacterial loads in symptomatic versus asymptomatic patients. The mean difference was 1.6 × 10^5^ copies/ml (95% CI − 1.4 × 10^5^–4.8 × 10^5^, p = 0.30) for women and 1.4 × 10^6^ copies/ml (95% CI -4.0 × 10^5^–3.2 × 10^6^, p = 0.12) for men.

**Conclusions:**

The cure rate of doxycycline in this study is higher than previously reported. This adds support to doxycycline’s role in treatment before initiating treatment with less favorable drugs such as moxifloxacin.

## Background

*Mycoplasma genitalium* is a sexually transmitted pathogen that can cause acute urethritis in men. It is associated with cervicitis and pelvic inflammatory disease (PID) in women, although less is known about its role in female reproductive tract syndromes[1, 2]. It has been associated with an increased risk of PID, infertility and adverse pregnancy outcomes [2, 3]. However, due to variable diagnostic criteria and the need for invasive diagnostic sampling the evidence to support these associations are weak.

Treatment of *M. genitalium* has become increasingly difficult. First-line treatment for *M. genitalium* in Europe was until recently the macrolide antibiotic azithromycin, either as a 1 g single-dose or as a 5-day regimen with 500 mg on day 1 and 250 mg on days 2–5[4]. The latter regimen has been recommended to avoid selection of macrolide resistance [5, 6]. Over the last few years, studies indicating high rates of treatment failure and increasing macrolide resistance has emerged all over the world. A meta-analysis evaluating the efficacy of 1 g azithromycin showed a pooled efficacy of 85.3% in studies performed prior to 2009 and 67.0% in studies performed between 2009 and 2013, indicating that azithromycin has become less effective over time [7]. In Trondheim, Norway, the number of macrolide resistant strains in clinical samples in 2017 was 73% (unpublished data). The implementation of a macrolide resistance test before treatment has been strongly advised in order to avoid unnecessary use of azithromycin where this is futile [8].

The current recommended treatment for macrolide resistant *M*. *genitalium* in Europe is moxifloxacin 400 mg daily for 7–10 days [4]. Moxifloxacin is a fluoroquinolone, an antibiotic drug that targets DNA gyrase and topoisomerase IV in bacterial DNA, inhibiting replication [9]. Adverse events described for this drug includes reduction of seizure threshold, QT prolongation, tendinopathy, arthropathy, neuropathies, liver toxicity, psychosis, and photosensitivity [10]. Several cases of fluoroquinolone resistance have been described, especially in Australia and Asia [8, 11, 12]. A review of antimicrobial resistance in Europe based on literature published between 2012 and 2018 report fluoroquinolone resistance-associated mutations in the parC gene with an estimated prevalence of 5% [13]. In Scandinavia, a study from 2017 reported a prevalence of fluoroquinolone resistance-associated mutations of 5.1% in Denmark, 4.1% in Norway and 10.2% in Sweden [14].

A meta-analysis evaluating treatment efficacy of moxifloxacin on *M. genitalium* infections showed a decrease in elimination rate from 100% on participants enrolled in studies prior to 2010 to 89% in participants enrolled in studies between 2010 and 2016[15]. The 2016 European Mycoplasma Guidelines suggest two third-line treatment options in cases of macrolide and fluoroquinolone treatment failures [4]. The first is pristinamycin, a streptogramin inhibiting protein synthesis, with a prescribed regime of 1 g four times daily for 10 days. The alternative third-line treatment is doxycycline 100 mg twice daily for 14 days. Doxycycline is a second-generation tetracycline, a bacteriostatic antibiotic acting on the ribosomal protein synthesis unit [16]. Benefits include good availability worldwide, affordability and good tolerability by most patients. Randomized controlled trials have found an efficacy of doxycycline of 30–45% for treatment of *M.genitalium* [17–20]. Thus far, no mutations associated with tetracycline resistance has been identified for *M. genitalium* and the reason for the low efficacy of tetracyclines is unknown.

Recently, Read et al. found a ≥ 92% cure rate of *M. genitalium* infections after conducting sequential antimicrobial therapy [21]. Patients presenting with nongonococcal urethritis, cervicitis or proctitis were initially treated with doxycycline 100 mg twice daily for 7 days followed by *M. genitalium* macrolide resistance testing, and subsequent resistance-guided therapy with either azithromycin 2.5 g (1 g on day 1 and 500 mg on days 2–4) if resistance negative or sitafloxacin 100 mg twice daily for 7 days if resistance positive. The pre-treatment therapy with doxycycline was assumed to lower bacterial load and thus likely contributed to the high cure rate. The sequential therapy was later evaluated with the more commonly used second-line drug moxifloxacin instead of sitafloxacin, with equal results [22]. The updated guidelines for management of *M. genitalium* in Britain, Australia and New Zealand now recommend a sequential therapy model for uncomplicated infection [23, 24].

### Aims

Following the implementation of macrolide resistance testing at St. Olav’s Hospital in Trondheim, Norway, in 2016, patients with macrolide resistant strains were treated with doxycycline 100 mg twice daily for 14 days to limit the use of moxifloxacin. This study aimed to evaluate the treatment efficiency of doxycycline on macrolide resistant *M. genitalium* infections. In extension, the study aimed to contribute to reducing unnecessary use of fluoroquinolones.

## Materials and methods

### Data collection

A list of patients testing positive for macrolide resistant *M. genitalium* from January 1st, 2016 to September 1st, 2019, was acquired. The samples originated primarily from the Department of Venereology at St. Olav’s Hospital, Trondheim, Norway. Additional samples originated from the Student Sexual Health Clinic at Gløshaugen Campus at the Norwegian University of Science and Technology, the Gynecology Department at St. Olav’s Hospital and the Youth Health Care Centre in Trondheim. The medical records for each of these patients were then accessed and information about treatment regime, control test results and symptom reports were gathered in a pre-defined spreadsheet. Only patients 16 years or above at the time of treatment, who received doxycycline 100 mg twice daily for 14 days, were included. All patients who had made a reservation from biological research were excluded. Other exclusion criteria included lack of test of cure (TOC) or a TOC taken > 3 months after the pre-treatment test. Once the list was complete, patients were de-identified. The final list of included patients consisted of 259 patients. Age, sex and symptom status are shown in Table 1.

**Table 1:** Patient characteristics, co-infections and treatment results

	Female	Male	Total
Patients	161 (62.2%)	98 (37.8%)	259
Infections	165 (62.7%)	98 (37.3%)	263
Median age, years (range)	21 (16–43)	24 (18–43)	22 (16–43)
Concurrent infection			
*Chlamydia trachomatis*	24 (14.5%)	15 (15.3%)	39 (14.8%)
*Neisseria gonorrhoeae*	1 (0.6%)	0	1 (0.4%)
Negative test of cure	112 (67.9%)	43 (43.9%)	155 (58.9%)
Positive test of cure	53 (32.1%)	55 (56.1%)	108 (41.1%)
Symptoms at inclusion*	67 (25.5%)	41 (15.6%)	108 (41.1%)
Positive test of cure	24 (35.8%)	25 (61.0%)	49 (45.4%)
Negative test of cure	43 (64.2%)	16 (39.0%)	59 (54.6%)

*Any report of symptoms of a sexually transmitted infection from the patient’s medical records

### Laboratory methods

Before inclusion in the study, all samples had undergone routine microbiological testing for sexually transmitted infections at the Department of Medical Microbiology at St. Olav’s Hospital. Swab samples were collected with flocked swabs in 2.0 ml UTM medium (Copan, Brescia, Italy). 1 mL of urine and 200 µL of transport medium from swab samples were extracted using the NucliSens EasyMag system (bioMerieux SA, Marcy l’Etoile, France), yielding 55 µl of DNA eluate. Polymerase chain reaction was performed using a CFX96 Real-Time PCR instrument (Bio-Rad Laboratories Inc., Hercules, CA, USA). The commercially available screening kit FTD Urethritis basic (Fast-track diagnostics Ltd, Esch-sur-Alzette, Luxembourg) was used for the simultaneous testing of *Neisseria gonorrhoeae*, *Chlamydia trachomatis* and *M. genitalium*. Any *M. genitalium* positive sample succeedingly underwent macrolide resistance testing using an in-house SimpleProbe PCR Assay [25]. The SimpleProbe assay was introduced as part of routine diagnostics at the laboratory in 2016 and has proven to be a reliable test, with a sensitivity of 95.2% in 2019 (unpublished data). In addition, quantification of all specimens positive for *M. genitalium* was performed in accordance with instructions from the manufacturer.

### Statistics

The sample size was calculated as follows: to obtain a 95% Wilson Score confidence interval with width 0.1 we required a sample size between 320 and 381 for a proportion between 0.3 and 0.5. Expanding the width to 0.2 lowered the required sample size to between 78 and 93 for a proportion between 0.3 and 0.5. The sample size calculation was carried out in the software PASS 2019, v19.0.2. The t-test was used to calculate differences in bacterial load. The analyses were performed using SPSS (IBM SPSS Statistics Version 26, International Business Machines Corporation). The remaining statistical analyses were performed using Microsoft Excel (Microsoft Corporation).

## Results

**Fig. 1:**
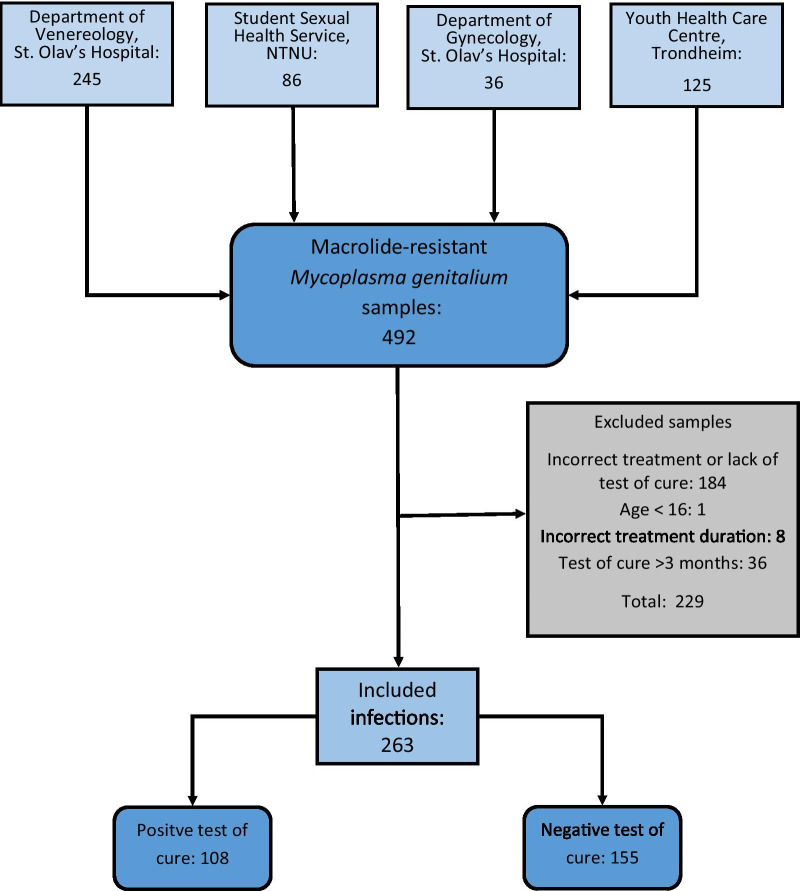
Distribution of patient samples. Sample inclusion and exclusion and result of test of cure following treatment.

259 patients were included in the study, four of whom had two separate *M. genitalium* infections during the inclusion time period. Figure 1 shows the distribution of the patient samples. Of the 263 infections evaluated, 155 (58.9%) had a negative TOC after two weeks of treatment with doxycycline 100 mg twice daily. Median time to TOC from the initial positive test based on available dates was 34 days (range 15–92 days). 229 patients from the original list of patients with macrolide resistant samples did not meet the inclusion criteria and were excluded from the study. The mean bacterial load for the 263 included infections was 8.4 x 105 copies/ml (SD 2.7 x 106 copies/ml). The mean difference in bacterial load between asymptomatic and symptomatic patients, as well as the mean difference in bacterial load between patients with a negative TOC and those with a positive TOC, are shown in Table 2.

**Table 2:** Bacterial load in the initial pre-treatment samples among male and female patients

	Bacterial load	
	Female	Male
Symptomatic, mean (range)	2.8 × 10^5^ copies/ml (764–3.8 × 10^6^ copies/ml)	1.1 × 10^6^ copies/ml (401–1.1 × 10^7^ copies/ml)
Asymptomatic, mean (range)	4.4 × 10^5^ copies/ml (383–6.4 × 10^6^ copies/ml)	2.5 × 10^6^ copies/ml (1.3 × 10^3^–3.2 × 10^7^ copies/ml)
Mean difference mean (95% CI, p)	1.6 × 10^5^ copies/ml (− 1.4 × 10^5^–4.8 × 10^5^ copies/ml, p = 0.30)	1.4 × 10^6^ copies/ml (− 4.0 × 10^5^–3.2 × 10^6^ copies/ml, p = 0.12)
Negative TOC, mean (range)	3.5 × 10^5^ copies/ml (383–6.4 × 10^6^ copies/ml)	7.8 × 10^5^ copies/ml (401–6.6 × 10^6^ copies/ml)
Positive TOC, mean (range)	4.2 × 10^5^ copies/ml (1.6 × 10^3^–4.4 × 10^6^ copies/ml)	2.3 × 10^6^ copies/ml (1.3 × 10^3^–3.2 × 10^7^ copies/ml)
Mean difference, mean (95% CI, p)	6.9 × 10^4^ copies/ml (− 2.5 × 10^5^–3.9 × 10^5^ copies/ml, p = 0.67)	1.5 × 10^6^ copies/ml (− 1.5 × 10^5^–3.2 × 10^6^ copies/ml, p = 0.07)

Table 3 shows the distribution of symptoms stratified by sex. 54.0% of the infections studied were asymptomatic and 4.9% had unknown symptom status. Of the 58.9% with a negative TOC, 57.4% were asymptomatic. Of the 49 patients who reported symptoms at inclusion that had not achieved microbiological cure at TOC, 17 (34.7%) had records of symptom improvement or clearance at the follow-up visit. 16 (32.7%) had no relief of symptoms and 16 (32.7%) had no information on changes in symptom status in their medical records.

**Table 3:** Self-reported symptom distribution before treatment, as noted in the patient’s medical records (n = 263)

	Female	Male	Total
Asymptomatic	91 (55.2%)	51 (52.0%)	142 (54.0%)
Symptoms present	67 (40.6%)	41 (41.8%)	108 (41.1%)
Dysuria	19 (11.5%)	32 (32.7%)	51 (19.4%)
Frequent urination	4 (2.4%)	1 (1.0%)	5 (1.9%)
Discharge*	38 (23.0%)	19 (19.4%)	57 (21.7%)
Itching	11 (6.7%)	9 (9.2%)	20 (7.6%)
Abdominal or pelvic pain/discomfort	6 (3.6%)	0	6 (2.3%)
Vaginal bleeding**	9 (5.5%)	NA	9 (3.4%)
Throat discomfort	2 (1.2%)	0	2 (0.8%)
Not stated	7 (4.2%)	6 (6.1%)	13 (4.9%)

*Discharge includes any change in discharge such as increased amount, odor, color and consistency

**Vaginal bleeding includes any abnormal bleeding, such as post-coital bleeding, bleeding between periods and bloody discharge

## Discussion

Increasing macrolide resistance has enforced alternative treatments for *M. genitalium* infections. Macrolide resistance testing before treatment allows for more appropriate management and contributes to reducing unnecessary use of macrolides where this is futile. Moxifloxacin, as the current recommended second-line treatment option in case of macrolide resistance, belongs to a class of antibiotics shown to be among the worst environmental contaminators [26]. Due to the long list of interactions, precautions and contra-indications of moxifloxacin it would be beneficial to limit its use. In this study, we report a 58.9% cure rate after a two-week course of doxycycline on macrolide resistant infections of *M. genitalium*. Although low relative to the effects shown for moxifloxacin, this longer course of doxycycline showed a higher efficacy than previously reported.

### Symptoms and bacterial load

Several patients in this study experienced a relief of symptoms or symptom clearance despite bacterial persistence. Doxycycline is thought to reduce bacterial load, and several studies have shown that *M. genitalium* infections with low bacterial loads are more likely to be eliminated [27, 28]. It is therefore possible that the included patients with low bacterial load were more likely to be cured. In our material, the pre-treatment bacterial load for patients with a negative TOC did not differ statistically from those with a positive TOC, although for men the difference was close to significant with a p-value of 0.07. We had no information on the duration of symptoms before the initial testing, a factor that is likely to influence the bacterial load. Without this information further analysis on differences in bacterial load between asymptomatic and symptomatic patients with or without a negative TOC becomes speculative. Available dates for TOC showed a median time of 34 days, with a range from 15 to 92 days. Due to the retrospective study design, there was a lack of standardization which makes exact time of treatment start impossible to assess. TOC was thus recorded as time from the initial pre-treatment test and not the day of treatment start. Even if PCR is a very sensitive test, detecting bacterial loads below 400 copies/ml, the early negative TOCs might represent false negative tests with bacterial loads below the detection limit, and thus overestimate the cure rate. This risk declines if TOC is taken at a later time, and many recommend performing TOC no earlier than 3weeks after treatment. However, the risk of reinfection increases with time. A study comparing one and two weeks of doxycycline with a standardized time to TOC would be beneficial.

Many patients had to be excluded from the study due to a lack of TOC. Whether these patients obtained a TOC or not is unknown. It is plausible that patients not experiencing symptoms at all or patients who experienced a relief of symptoms following treatment were less likely to return for TOC than those with persistent symptoms. Data regarding TOC were inconsistent in the journal notes and advise not to return for a control test when asymptomatic increased from 2019. In addition, advice not to test asymptomatic individuals has increased over the last years, as research has failed to provide clear evidence of severe outcome if *M. genitalium* is left untreated [2, 29, 30].

### Strengths and limitations

Evaluations of new treatments are ideally performed as randomized controlled trials (RCTs) to minimize risk of bias and to control variables. A retrospective observational study like ours has limitations but provides insight into clinical management and adherence of local guidelines with valuable data to support the design of possible prospective studies. 259 patients were included in our study. The sample size was initially estimated to obtain a 95% confidence interval with a width of 0.1. Estimated treatment effect was 30–50%, based on previous studies [17, 19, 31] and preliminary estimates from clinical practice. Although slightly lower than we aimed for, the total amount of included infections is high, providing an acceptable external validity. The main limitation to a retrospective study design is the inability to control which information is available in the medical records and the inability to control for appropriate follow-up. In our study, this resulted in a very high number of patients not meeting inclusion criteria due to lack of TOC within reasonable time. For study purposes a TOC within three months was regarded appropriate. Many had control tests taken later than this and were thus excluded because the risk of re-infection could not be ruled out. Also, spontaneous microbiological clearance is thought to happen, and the likelihood increases with time [32]. Negative control tests taken later than 3 months after treatment can thus be the result of spontaneous clearance rather than the treatment. Many of the TOCs were self-tests and thus the records contained no information of symptoms. As a consequence of the retrospective study design, no instructions were given to health care professionals in advance, and medical notes and patient guidance varied both between and within study sites. It would have been relevant to include all positive *M. genitalium* samples from the laboratory for the evaluation. However, the ethical approval obtained gave permission to use the macrolide resistant samples only.

## Conclusions

Microbiological cure following treatment with doxycycline 100 mg twice daily for two weeks was found in 58.9% of 263 M*. genitalium* infections in a Norwegian population between January 2016 and September 2019. This is higher than previously reported. Despite several limitations to the study design, our study adds support to doxycycline’s role in treatment of macrolide-resistant *M. genitalium* before initiating treatment with less favorable drugs like fluoroquinolones.

## Data Availability

The data sets used and analyzed during the current study are not publicly available due to privacy of the included patients but are available from the corresponding author on reasonable request.

## Abbreviations

FVU: First-void urine
M. genitalium: *Mycoplasma genitalium*
RCT: Randomized controlled trial
TOC: Test of cure
